# lncRNA-HEIM Facilitated Liver Fibrosis by Up-Regulating TGF-*β* Expression in Long-Term Outcome of Chronic Hepatitis B

**DOI:** 10.3389/fimmu.2021.666370

**Published:** 2021-06-08

**Authors:** Jian Yao, Chenhong Lin, Jingjing Jiang, Xujun Zhang, Fengxia Li, Tianxing Liu, Hongyan Diao

**Affiliations:** ^1^ State Key Laboratory for Diagnosis and Treatment of Infectious Diseases, National Clinical Research Center for Infectious Diseases, Collaborative Innovation Center for Diagnosis and Treatment of Infectious Diseases, The First Affiliated Hospital, College of Medicine, Zhejiang University, Hangzhou, China; ^2^ Department of Biological Sciences, University of Toronto, Toronto, ON, Canada

**Keywords:** chronic hepatitis B virus (HBV), long-term antiviral treatment, lncRNA, monocyte, transforming growth factor *β* (TGF-*β*), liver fibrosis

## Abstract

**Background:**

Chronic liver fibrosis is an inevitable stage for the development of patients with chronic hepatitis B (CHB). However, anti-fibrotic therapies have been unsuccessful so far. The biological functions and molecular mechanisms of long non-coding RNAs (lncRNAs) in the host immune system during chronic hepatitis B virus (HBV) infection, especially in fibrosis, are still largely unknown.

**Method:**

The total RNA of peripheral blood mononuclear cells (PBMCs) from asymptomatic carriers (ASCs) or CHB receiving at least 8 years of anti-viral treatments was analyzed using Arraystar microarray and validated *via* quantitative real-time PCR (qRT-PCR). Correlation analysis was conducted based on correlation coefficients, Clusterprofile, and RNA Interactome Database (RAID). The functions of lncRNA in monocytes were determined *via* loss-of-function RNAi or gain-of-function lentivirus assays. The expression levels of mRNAs or proteins were evaluated using qRT-PCR, western blotting assay, or enzyme linked immunosorbent assays (ELISA).

**Results:**

A total of 1,042 mRNA transcripts (630 up-regulated and 412 down-regulated) were identified being differentially expressed between ASC and CHB patients. Through enrichment analysis we focused on the transforming growth factor beta (TGF-*β*) signaling pathway and validated their expression in a larger cohort. Moreover, we found that lncRNA ENST00000519726 (lncRNA-HEIM) was highly expressed in monocytes and further up-regulated upon HBV infection. LncRNA-HEIM played an important role in CHB patients with long-term antiviral treatments, and its elevated expression was remarkably correlated with the TGF-*β* signaling pathway, especially with the two members namely TGF-*β* and SMAD4. Furthermore, altering the endogenous lncRNA-HEIM level in monocytes significantly affected the production of TGF-*β*, as well as the fibrosis of hepatic stellate cells by affecting the expression of collagen I and *α*-smooth muscle actin (*α*-SMA).

**Conclusion:**

These findings not only added knowledge to the understanding of the roles of which lncRNA-HEIM played in the activation of HSCs in CHB patients with long-term medication, but also provided a promising therapeutic target in the future treatment for liver fibrosis.

## Introduction

Viral hepatitis was the seventh leading cause of mortality worldwide and responsible for 1.4 million deaths per year (1). Especially, chronic infections of hepatitis B virus (HBV) can lead to immune mediated liver damage which could further progress to cirrhosis and hepatocellular carcinoma (HCC) and is still incurable because of the persistence of a covalently closed circular DNA (cccDNA) of HBV in hepatocytes during the infection (2). As a common outcome of chronic HBV infection, the patients with progressive liver fibrosis are at risk of developing into cirrhosis and liver cancer, and blocking the process of liver fibrosis becomes the key in treating chronic HBV infections (3). Despite the emerging non-invasive methods including serum biomarker algorithms and transient elastography assessments, percutaneous liver biopsy remained as the gold standard for the staging of fibrosis for its advantage in viability, accuracy, and risk factors for error (4). Although a considerable number of therapeutic targets in liver fibrosis have been identified, clinical trials of anti-fibrotic therapies have been unsuccessful so far (5). Non-invasive methods which are accurate and reliable for the diagnosis of early liver fibrosis are urgently needed for the treatment of CHB patients, as well as therapies that could effectively block the progression of fibrosis.

The chronic HBV infection is a complex process, involving diverse factors and interactions between the host immune system and the virus (6). As the first step in the cascade of malignant progression, liver fibrosis is preceded by inflammation, where elements of both the innate and adaptive immune systems are pivotal in its regulations. Among these immune cells, macrophages exert critical functions ranging from eliminating pathogens to maintaining immunological tolerance, as well as initiating and perpetuating inflammation in response to injury, promoting liver fibrosis through activating hepatic stellate cells, and resolving inflammation and fibrosis by degrading extracellular matrix and releasing anti-inflammatory cytokines (7). The cellular heterogeneity of hepatic macrophage and its central role in the pathogenesis of chronic liver injury made it a promising potential target in combatting fibrosis.

Recently, accumulating reports have suggested the existence of a novel class of RNA named long non-coding RNAs (lncRNAs), which is defined as transcripts with longer than 200 nucleotides in length but without protein translation capability. The expression pattern of lncRNAs can be highly specific among cells or tissues, and the dysregulation of lncRNAs is also significantly correlated with different diseases, including multiple cancers (8–10). As to viral-infectious diseases, more and more research attention was paid to the functions of lncRNAs in the host immune response against viral infection (11). Hao et al. performed microarray on liver tissues and identified a total of 203 differentially expressed lncRNAs in patients with chronic HBV infection, most of which might be involved in cytokine–cytokine receptor interaction and varied biotransformation processes including fatty acid metabolism, amino acid metabolism, carbon metabolism, and drug metabolism (12). Nevertheless, the biological functions as well as the molecular mechanisms of lncRNAs in the host immune system during HBV infection still remain largely unknown.

In this study, we identified an unreported lncRNA ENST00000519726 with high expression in monocytes and named it as lncRNA Highly Expressed In Monocytes (lncRNA-HEIM). The expression of lncRNA-HEIM could be up-regulated in the monocytes of CHB patients with long-term antiviral treatment, leading to the subsequent elevation of TGF-*β* and SMAD4. The increased expression of TGF-*β* could further promote the fibrosis of hepatic stellate cell. These findings expand the understanding of lncRNA’s role in the immune response of long-term CHB patient, providing promising targets for the future research and treatments on patients with chronic HBV infection.

## Materials and Methods

### Ethics and Blood Sample Collection

Human blood samples from asymptomatic carrier (ASC) or chronic hepatitis B (CHB) patients or healthy volunteers were obtained from donors with their informed consent. This study and the usage of these blood samples were approved by the Ethics Committee of the First Affiliated Hospital, College of Medicine, Zhejiang University (Ethics number: 2018-970). Fifteen healthy volunteers (seven females and eight males) were randomly recruited from those who visited the First Affiliated Hospital of Zhejiang University for their physical examination at 2018 and 2021, with an average age of 37.9 (medium age 38, range 28–47). A total of twelve CHB patients and eighteen ASC patients were recruited from the First Affiliated Hospital, College of Medicine, Zhejiang University, between 2017 and 2018. CHB patients had received an anti-viral therapy of entecavir for eight years, with major hepatic functions like aspartate aminotransferase (AST) and normal alanine aminotransferase (ALT) levels. No observable radiological liver fibrosis was found in CHB patients or ASC patients. The summarized patient information was listed in **Supplementary Table S1**.

### RNA Preparation and Quantification of PBMCs

About 5 ml blood samples were collected, and the PBMCs were isolated using standard density-gradient centrifugation on Ficoll-Paque [MultiSciences (Lianke) Biotech, China]. In particular, CD14^+^ monocytes were isolated from PBMCs of either healthy donors or CHB patients using EasySep human CD14 positive selection kit II (Stemcell Tech, Cambridge, MA) following the manufacturer’s instruction. In brief, PBMCs were resuspended at 1 × 10^8^ cells/ml in EasySep buffer. Isolation cocktail mix and magnetic particles from the kit were added into cells, and a series of incubation and centrifugation steps were performed. Finally, CD14^+^ monocytes were isolated, and the rest were CD14^−^ cells. CD19^+^ B lymphocytes, CD3^+^ T lymphocytes, and natural killer (NK) cells were isolated from the PBMCs of HDs using EasySep human CD19 positive selection kit II (Stemcell Tech), EasySep human CD3 positive selection kit II (Stemcell Tech), and EasySep human NK Cell isolating kit (Stemcell Tech) respectively, following the manufacturer’s instruction. Total RNA was isolated using RNAiso Plus (Takara Bio, Dalian, China) according to the manufacturer’s instructions. The concentration of RNA was determined by Nanodrop One (Thermo-Fisher Scientific, MA) and diluted using DEPC-treated water. RNA was transcripted into cDNA using PrimeScript RT Master Mix (Perfect Real Time) (Takara Bio, Dalian, China), and quantitatively amplified on QuantStudio 5 (Applied Biosystems, CA) using SYBR Premix Ex Taq II (Perfect Real Time) (Takara Bio, Dalian, China), following the manufacturer’s instructions. All the primers for quantitative PCR (shown in **Supplementary Table S2**) were synthesized by Sangon Biotech (Shanghai). Data were normalized using glyceraldehyde 3-phosphate dehydrogenase (GAPDH) as internal controls and calculated using the 2^−ΔΔCT^ method.

### Microarray Analysis

Sample preparation and microarray hybridization of PBMC RNA of ASC or CHB patients with long-term antiviral treatments were conducted by KangChen Bio‐tech (Shanghai, China). Generally, lncRNAs and mRNAs were purified from total RNA after removal of rRNA (mRNA-ONLY™ Eukaryotic mRNA Isolation Kit, Epicentre). Then, each sample was amplified and transcribed into fluorescent cRNA along the entire length of the transcripts without 3′ bias utilizing a random priming method (Arraystar Flash RNA Labeling Kit, Arraystar). Each labeled cRNA was fragmented and hybridized on Human LncRNA Microarray V4.0 (Arraystar, containing 40,173 annotated lncRNAs or lncRNAs of high confidence, as well as an entire collection of 20,730 protein coding mRNAs). After washing, the hybridized arrays were scanned using the Agilent DNA Microarray Scanner. Data processing was performed with the GeneSpring GX v12.1 software package (Agilent Technologies), and reads of high quality or with raw intensity more than 40 were chosen for further analysis. Differentially expressed mRNAs and lncRNAs with statistical significance between the two groups were identified through P-value/FDR filtering and fold change filtering, with the cutoff for P-value/FDR at <0.01 and for fold change >2 (up- or down-regulated). KEGG pathway analysis was applied to determine the roles of these differentially expressed mRNAs played in these biological pathways. Hierarchical clustering and combined analysis were performed using in-house scripts. The correlation analysis between lncRNAs and mRNAs was conducted as follows. The up-regulated mRNAs were subjected to KEGG annotation using R package namely ClusterProfile. Candidate correlated lncRNAs of which the interaction score with target gene was >0.95 based on RNA Interactome Database (RAID) v 3.0, as well as the Pearson correlation coefficient | cor | >0.7, were selected for further analysis. NetworkD3 and Hmise R packages were used to depict the alluvial diagram of the correlation between lncRNAs, mRNAs and corresponding KEGG pathways.

### Cell Culture

Human monocyte cell line THP-1 and human hepatic stellate cell line LX-2 were from the State Key Laboratory for Diagnosis and Treatment of Infectious Diseases. The cells were cultured in either RPMI-1640 (for THP-1) or high glucose DMEM (Dulbecco’s modified Eagle’s medium, for LX-2) supplemented with FBS (fetal calf serum) to a final concentration of 10% and antibiotics at 37℃ with 5% CO_2_. THP-1 cells were stimulated with 100 ng/ml PMA (Phorbol-12-myristate-13-acetate) for 24 h unless indicated otherwise. LX-2 cells were seeded into a corning costar transwell plate (100,000 cells per well) in a co-culture system with THP-1 cells.

### Oligonucleotide Transduction

Small interfering RNAs (siRNAs) targeting lnc-HEIM were designed and synthesized by GenePharma (Shanghai, China), of which the sequences are listed in **Supplementary Table S3**. Cells were transfected with 60 μM siRNAs at 50% confluence using Lipofectamine 2000 (Invitrogen) and harvested for analysis or co-culture assays 24 h after transfection.

### Lentivirus Infection

Recombinant lentiviral particles expressing lncRNA-HEIM or negative control were obtained from Hangzhou Baixi Biotech (Hangzhou, China). THP-1 cells were infected with lentivirus at multiplicity of infection (MOI) of 50 for 24 h, together with 10 μg/ml polybrene (Genechem, Shanghai, China). Cells stably expressing lncRNA-HEIM or negative control were selected with blasticidin S HCl (Gibco, USA). The expressing levels were determined *via* qRT-PCR.

### Western Blot Analysis

Cell lysates were resolved *via* SDS-PAGE, transferred to nitrocellulose membrane (Bio-Rad, Hercules, CA), and blocked in PBS/Tween-20 containing 5% non-fat milk. The membrane was incubated with antibodies for *α*-SMA (Abcam, ab32575, Cambridge, UK), collagen I (Santa Cruz, sc-166865), TGF-beta1 (Abcam, ab92486, Cambridge, UK), Phospho-Smad3 (Cell Signaling Technology, #9520, Beverly, Massachusetts, USA), Phospho-SMAD2(Cell Signaling Technology, #18338), Smad2/3 (Cell Signaling Technology, #8685), Smad4 (Cell Signaling Technology, ab40759), and GAPDH (Huabio, EM1101, Hangzhou, China). Immunoreactive proteins were visualized using ECL detection system (Millipore, Bedford, MA, USA). The chemiluminescent density was determined using ImageJ 1.52a, and normalized with GAPDH. Fold changes were indicated in the figure based on the negative control, or indicated otherwise in the corresponding figure legend.

### Cytoplasmic and Nuclear RNA Purification

Cytoplasmic and nuclear RNAs were isolated and purified using RNeasy midi kit (Qiagen, Valencia, CA) following the manufacturer’s instruction. Briefly, cells were lysed using RLN on ice, and the lysates were centrifuged to separate the nucleus from cytoplasm. Cytoplasmic or nuclear RNAs were purified respectively through RNeasy spin columns and resolved in DEPC water. Relative RNA expression levels were determined *via* qRT-PCR.

### Confocal Microscopy

LX-2 cells were seeded on Poly-lysine-pre-coated glass cover slips and incubated with THP-1 cells stably over-expressing lncRNA-HEIM or mock control in transwell (3.0 μm, Corning Costar, USA) for 36 h. After washing with PBS, cells were sequentially fixed with 4% ice-cold paraformaldehyde for 30 min, permeabilized with 1% Triton X-100 for 20 min, and blocked using 5% bovine serum albumin (BSA) in PBS for 1 h at room temperature. Next, cells were incubated with primary antibodies against *α*-SMA (1:100, rabbit, Abcam, ab32575), COL1A2 (1:100, mouse, Santa Cruze, sc-166865) overnight at 4°C, followed by incubation with FITC-conjugated secondary antibodies (1:150, Thermo Fisher, USA) and PE-conjugated secondary antibodies (1:150, Thermo Fisher, USA) in PBS away from light for 30 min at room temperature. And the nuclei were stained with DAPI for 20 min. All immunofluorescence was then visualized using confocal microscope (NIKON A1R).

### ELISA Assays

Commercial ELISA kit for TGF-beta1 (Invitrogen, Human/Mouse TGF beta 1 Uncoated ELISA # 88-50350, USA) was employed to measure the concentrations of these cytokines in patients and cell culture supernatant according to the manufacturer’s instructions.

### Statistical Analysis

Statistical analyses were performed using GraphPad Prism software (version 7.0). Comparisons over groups were analyzed using two-way ANOVA followed by Tukey’s multiple comparisons test, and comparisons of multiple samples at one group were analyzed using two-tailed t test. The results were presented as the mean ± standard deviation values (SDs). In all figures with error bars, data are presented as mean ± SD. In all figures, N.S., not significant, p > 0.05; ∗p < 0.05; ∗∗p < 0.01; ∗∗∗p < 0.001; ∗∗∗∗p < 0.0001.

## Results

### RNA Profiling in Long-Term Chronic Hepatitis B Patients and Asymptomatic Carrier

**Figure 1:**
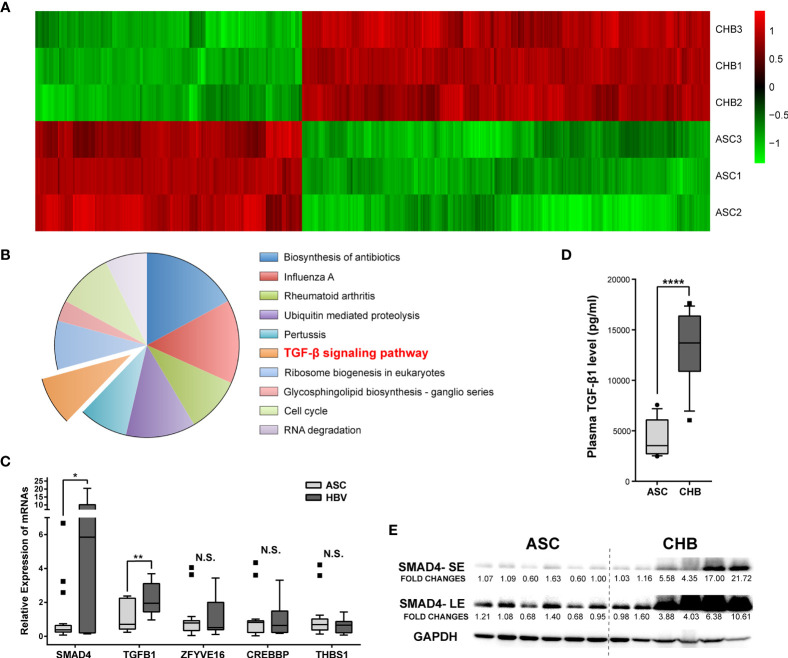
Expression differences and enrichment analysis of mRNAs in asymptomatic carrier (ASC) *vs.* chronic hepatitis B (CHB) samples with long-term antiviral treatment. **(A)** Hierarchical clustering analysis of 1,042 differentially expressed mRNA transcripts between CHB with long-term antiviral treatment and ASC samples (fold change >2, up- or down-regulated). Columns represent each gene, and rows represent each sample. The relative expression levels of mRNAs are depicted into color scale ranging from green (down-regulated) to red (up-regulated). **(B)** Pie charts of top ten enriched KEGG pathways including 630 up-regulated mRNA transcripts. **(C)** Quantitative real-time PCR validation of de-regulated genes from TGF-*β* signaling pathway in 18 ASC or 12 CHB samples. N.S., not significant, p > 0.05; ∗p < 0.05; ∗∗p < 0.01. **(D)** ELISA based serum levels of TGF-*β* in ASC or CHB samples. ∗∗∗∗p < 0.0001. **(E)** Western blotting analysis of SMAD4 protein levels in ASC or CHB samples. SE, short-time exposure; LE, long-time exposure. Chemiluminescent densities were normalized with GAPDH, and fold changes were calculated based on the average densities of ASC groups.

The PBMC transcriptomic profiles including both messenger RNAs (mRNAs) and long non-coding RNAs (lncRNAs) were obtained from the CHB patients with long-term antiviral treatment or ASC through microarray Arraystar LncRNA. Comparing with the ASC, we identified a total of 1,042 mRNAs transcripts (630 up-regulated and 412 down-regulated, respectively) differently expressed in CHB patients (**Figure 1A**). Resorting to Kyoto Encyclopedia of Genes and Genomes (KEGG) enrichment analysis, we focused on the up-regulated mRNAs and found that TGF-*β* (transforming growth factor beta) was the only related signaling pathway among the top ten most enriched ones (**Figure 1B**).

As an important cytokine, TGF-*β* played critical roles in the responses against viral infections (13), as well as in hepatic fibrosis and development of cirrhosis (14). Since TGF-*β* signaling pathway is activated in patients with chronic HBV infection (15), we verified the involving de-regulated genes in a larger cohort including 12 CHB patients with long-term antiviral treatment and 18 ASC. Results showed that SMAD4 and TGF-*β*1 genes were substantially up-regulated in CHB patients (**Figure 1C**). Particularly, the plasma TGF-*β* level, as well as the protein level of SMAD4 in the PBMC, was significantly elevated in CHB patients with long-term antiviral medication (**Figures 1D, E**), which was also in accord with the microarray results.

### Identification of Differentially Expressed lncRNAs Closely Related With TGF-*β* Signaling Pathway in Long-Term CHB Patients

**Figure 2:**
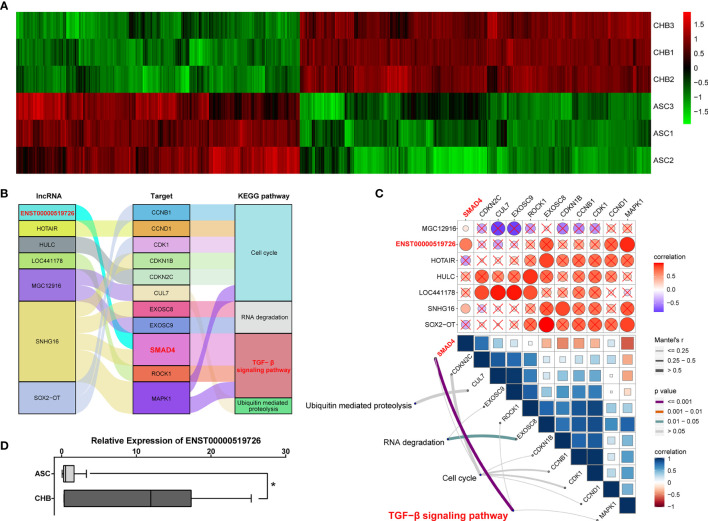
Identification of differentially expressed lncRNAs correlated with TGF-*β* signaling pathway in long-term CHB patients. **(A)** Hierarchical clustering analysis of 4,718 differentially expressed lncRNA transcripts between CHB with long-term antiviral treatment and ASC samples (fold change >2, up- or down-regulated). Columns represent each gene, and rows represent each sample. The relative expression levels of mRNAs are depicted into color scale ranging from green (down-regulated) to red (up-regulated). **(B)** Alluvial diagram of candidate differentially expressed lncRNAs with up-regulated mRNAs from enriched KEGG pathways. **(C)** Pairwise correlations between lncRNAs and mRNAs (upper), with a color gradient denoting Pearson correlation coefficients. Pairwise comparisons between lncRNAs and mRNAs (lower). Edge width corresponds to the Mantel’s *r* statistic, and edge color denotes the statistical significance. **(D)** Real-time PCR validation of lncRNA ENST00000519726 in ASC or CHB samples. ^∗^p < 0.05.

Although reports on the importance of lncRNAs during the hepatic tumorigenesis and further progression were increasing rapidly (16), their involvement in chronic hepatitis B is still largely unknown. In our microarray assay, a total of 4,718 lncRNA transcripts differentially expressed between ASC and long-term CHB patients had been identified, including 2,609 up-regulated and 2,109 down-regulated lncRNA transcripts (**Figure 2A**) To further illustrate their roles in chronic hepatitis B, we performed the correlation analysis between lncRNA transcripts and up-regulated mRNA using ClusterProfile and RNA Interactome Database (RAID v 3.0). Focusing on the top 10 up-regulated KEGG pathways shown in **Figure 1B**, we found that the TGF-*β* signaling pathway (13–15), cell cycle signaling pathway (17–19), RNA degradation pathway (20, 21), and ubiquitin mediated proteolysis pathway (22, 23) were broadly reported to be involved in the progress of viral infection including HBV infection. Therefore, we analyzed the correlated de-regulated lncRNAs with up-regulated mRNAs among these four KEGG annotated pathways, and found seven lncRNAs correlated with 11 target mRNAs (**Figure 2B**). Furthermore, gene set variation analysis (GSVA) indicated that only lncRNA ENST00000519726 was positively correlated with SMAD4 and TGF-*β* signaling pathway with statistically significant difference (**Figure 2C**). The following real-time PCR validation also showed that lncRNA ENST00000519726 was remarkably up-regulated in the long-term CHB patients (**Figure 2D**).

### lncRNA ENST00000519726 Was Highly Expressed in Monocyte

**Figure 3:**
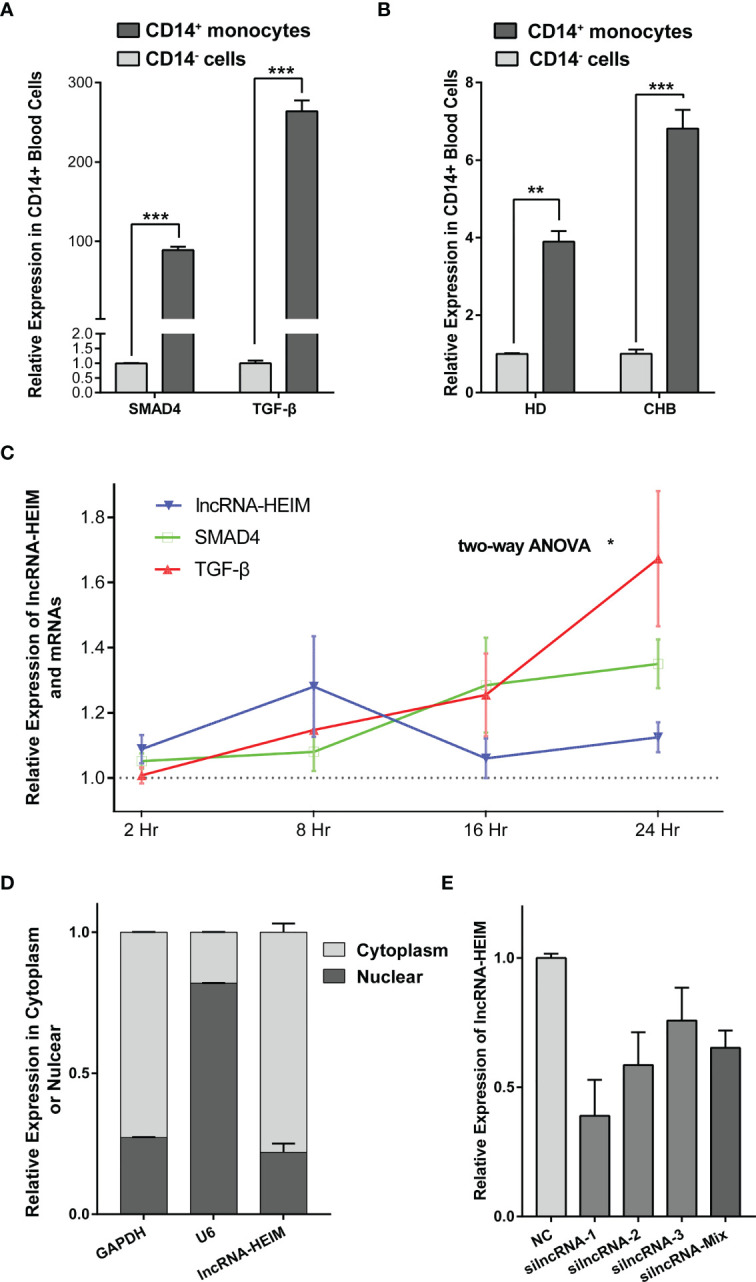
lncRNA-HEIM ENST00000519726 was highly expressed in monocytes. **(A)** Relative expression levels of SMAD4 and TGF-*β* in CD14^+^ monocytes against those in CD14^−^ cells from CHB patients. **(B)** Relative expression levels of lncRNA ENST00000519726 in CD14^+^ monocytes against those in CD14^-^ cells from either healthy donor (HD) or CHB patients. **(C)** Relative expression levels of lncRNA-HEIM, SMAD4, and TGF-*β* in monocytes at 2, 8, 16, and 24 h upon HBV infection. Expression levels of lncRNA-HEIM, SMAD4, and TGF-*β* were normalized with negative control at each time point. Comparisons over groups were analyzed using two-way ANOVA followed by Tukey’s multiple comparisons test. ^∗^p < 0.05. Blue line is for lncRNA-HEIM, while green and red lines are for SMAD4 and TGF-*β*, respectively. **(D)** Relative expression levels of lncRNA-HEIM, glyceraldehyde-3-phosphate dehydrogenase (GAPDH), and U6 in cytoplasm or nuclear of monocytes. GADPDH was used as cytoplasmic control transcripts, and U6 as nuclear control. **(E)** Relative expression levels of lncRNA-HEIM transfected with negative control or siRNAs targeting lncRNA-HEIM (silncRNAs). **p < 0.01, ***p < 0.001.

Recently, the importance of liver macrophages (including Kupffer cells and liver monocyte-derived macrophages) during hepatitis B infection has been increasingly valued. Their anti-inflammatory responses favored by HBV resulted in liver tolerance, activation of hepatic stellate cells, and subsequently HBV-associated liver pathologies including fibrosis, cirrhosis, and the progression to hepatocellular carcinoma (24). In light of these findings, we managed to isolate CD14^+^ monocyte cells from the PBMCs of CHB patients and examined the RNA levels *via* real-time PCR. As depicted in **Figure 3A**, the expression levels of TGF-*β*, SMAD4 was notably higher in CD14^+^ monocytes compared with the CD14^−^ cells. Interestingly, lncRNA ENST00000519726 was also highly expressed in CD14^+^ cells from PMBCs of either healthy donors (HDs) or CHB patients (**Figure 3B**). We further detected the expression of ENST00000519726 in CD14^+^ monocytes, CD19^+^ B lymphocytes, CD3^+^ T lymphocytes, and natural killer (NK) cells isolated from the PBMCs of HDs and found that its expression in CD14^+^ monocytes was much higher than the other lymphocytes (**Supplementary Figure 1**). Given these findings, this lncRNA ENST00000519726 in the following context was named as lncRNA Highly Expressed In Monocytes (lncRNA-HEIM).

### lncRNA-HEIM Promoted the Expression of TGF-*β* Upon Long-Term HBV Infection

In order to validate the correlation between lncRNA-HEIM expression and HBV infection, we co-cultured PMA-differentiated THP-1 macrophages with HepG2.2.1.5 supernatant, which contained intact HBV particles. Upon HBV infection, the expression of lncRNA-HEIM in THP-1 started to increase 2 h after the addition compared with negative control and reached climax at 8 h after. Even though dropped at 16 h, the relative lncRNA-HEIM level was still higher than that in negative control throughout the whole experiment. However, the fold changes of SMAD4 and TGF-*β* expression were less than that of lncRNA-HEIM within the first 8 h, until 16 h and later (**Figure 3C**). This indicated that lncRNA-HEIM might respond prior to TGF-*β* signaling pathway members upon HBV. As shown in **Figure 3D**, the sub-cellular location of lncRNA-HEIM was mainly in the cytoplasm, indicating its involved biological functions might occur at post-transcriptional levels.

**Figure 4:**
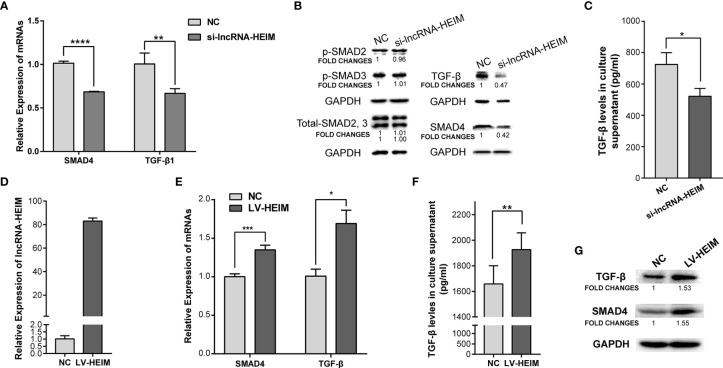
Over-expression of lncRNA-HEIM remarkably promoted the expression of TGF-*β* and SMAD4. **(A)** Relative expression of SMAD4 or TGF-*β* in monocytes transfected with siRNAs for lncRNA-HEIM or negative control (NC). ^∗∗^p < 0.01; ^∗∗∗∗^p < 0.0001. **(B)** Western blotting assays of endogenous protein levels of TGF-*β*, SAMD4, phosphorylated SMAD2, 3, and total SMAD2, 3 in monocytes transfected with siRNAs for lncRNA-HEIM or NC. GAPDH protein levels were used as internal controls. Chemiluminescent densities were normalized with GAPDH, and fold changes were calculated based on NC. **(C)** ELISA based TGF-*β* levels in culture supernatant of monocytes transfected with siRNAs for lncRNA-HEIM or NC. ^∗^p < 0.05. **(D)** Relative expression of lncRNA-HEIM in monocytes infected with lncRNA-HEIM lentivirus (LV-HEIM) or negative control (NC). **(E)** Relative expression of TGF-*β* or SMAD4 in monocytes stably over-expressing lncRNA-HEIM or NC. ^∗^p < 0.05; ^∗∗∗^p < 0.001. **(F)** ELISA based TGF-*β* levels in culture supernatant of monocytes with LV-HEIM or NC. ^∗∗^p < 0.01. **(G)** Western blotting assays of endogenous protein levels of TGF-*β*, SAMD4 in monocytes with LV-HEIM or NC. Fold changes, same as above.

To better understand the potential functions of lncRNA-HEIM in monocyte, the specific targeting siRNAs were designed and transfected in THP-1 monocytes, and the siRNA with the best silencing effect was used afterwards (**Figure 3E**). With the down-regulation of HEIM, the endocellular expression levels of TGF-*β* and SMAD4 were significantly reduced at both RNA and protein levels, while the protein levels of both phosphorylated SMAD2 and SMAD3 showed no obvious changes (**Figures 4A, B**). Accordingly, the TGF-*β* level in the culture supernatant was also notably down-regulated when transfected with HEIM siRNAs (**Figures 4C**), indicating that lncRNA-HEIM might play an important role in the TGF-*β* signaling pathway.

To further confirm the role HEIM played in monocytes, we constructed THP-1 cells which stably over-expressed lncRNA-HEIM *via* lentivirus. As demonstrated in **Figure 4D**, the average expressing level of HEIM in stable THP-1 cells (LV-HEIM) was about 80 folds compared with that in negative control cells (NCs). With the up-regulation of HEIM, the mRNA levels (**Figure 4E**) and the protein levels (**Figure 4G**) of TGF-*β* and SMAD4 rose correspondingly in LV-HEIM cells, which was consistent with the results in HEIM knock-down cells. Also, the up-regulation of the supernatant TGF-*β* level in LV-HEIM cells was of statistical significance (**Figure 4F**), which suggests that lncRNA-HEIM could prompt TGF-*β* signaling pathway by up-regulating the expression of both TGF-*β* and SMAD4.

### lncRNA-HEIM Remarkably Facilitated the Fibrosis of Hepatic Stellate Cells

**Figure 5:**
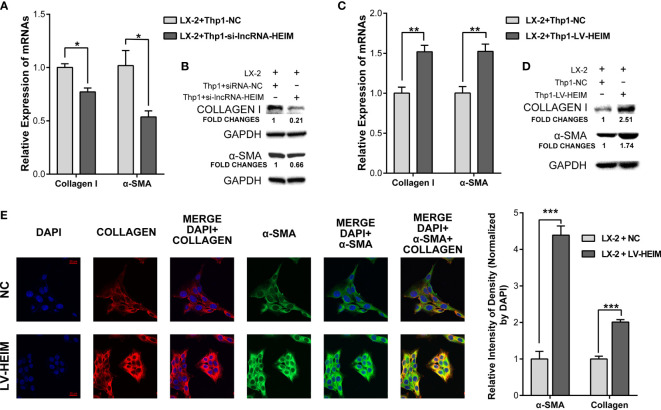
lncRNA-HEIM dramatically facilitated the fibrosis of hepatic stellate cells. **(A, B)** Relative mRNA expression and western blotting assays of collagen I and *α*-smooth muscle actin (*α*-SMA) in hepatic stellate cells (HSCs) co-cultured with monocytes transfected with siRNAs for lncRNA-HEIM or NC. Chemiluminescent densities in **(B)** were normalized with GAPDH, and fold changes were calculated based on NC. ^∗^p < 0.05. **(C, D)** Relative mRNA expression and western blotting assays of collagen I and *α*-smooth muscle actin (*α*-SMA) in HSCs co-cultured with LV-HEIM stable monocytes or NC. Fold changes in **(D)**, same as above. ^∗∗^p < 0.01. **(E)** Confocal imaging of collagen-I (red), *α*-SMA (green), and DAPI (blue) in HSCs co-cultured with LV-HEIM stable monocytes or NC. Scale bar: 20 μm. Relative intensity of density normalized by DAPI was depicted on the right. ^∗∗∗^p < 0.001.

The secretion of TGF-*β* by monocytes upon the infection of HBV could activate hepatic stellate cells *via* TGF-β1-CD147 loop and initiate HBV-associated fibrogenesis (25). To better illustrate the effect of lncRNA-HEIM in the process of liver fibrosis, we conducted the co-cultured model containing both THP-1 and LX-2 hepatic stellate cells. With the decreased TGF-*β* level due to the knock-down of HEIM in THP-1 cells, the mRNA levels of collagen-I and *α*-smooth muscle actin (*α*-SMA) were obviously reduced by about 30 and 50%, respectively (**Figure 5A**). The western blotting assay further confirmed that the protein levels of both collagen-I and *α*-SMA were decreased in LX-2 cells which were co-cultured with HEIM-siRNA transfected THP-1 cells (**Figure 5B**). On the contrary, while co-cultured with LV-HEIM cells, both the transcript and protein levels of collagen-I and *α*-SMA in LX-2 cells were substantially increased (**Figures 5C, D**). Resorting to immunofluorescence staining using confocal imaging, we again observed that co-culturing with LV-HEIM dramatically increased the protein levels of both collagen-I and *α*-SMA in LX-2 cells, which was in agreement with previous assays (**Figure 5E**).

## Discussion

In this study, we evaluated the transcriptional expression of both mRNAs and lncRNAs of CHB patients with long-term antiviral treatment. By focusing on the TGF signaling pathway, we identified an unreported lncRNA named ENST00000519726 (lncRNA-HEIM). The expression of lncRNA-HEIM could be up-regulated upon HBV infection, leading to the following rising expression of both TGF-*β* and SMAD4 and further facilitating the fibrosis of hepatic stellate cells. Collectively, our findings enrich the understanding of the immune response during the HBV chronic infection and shed light on the future treatment strategies by targeting lncRNAs.

It is widely accepted that chronic HBV persistent infection could cause the dysfunction of innate and adaptive immune responses. HBV infection could increase the secretion of TGF-*β* (26) and interleukin-10 (IL-10) (27) from monocyte/macrophage, while inhibit the secretion of tumor necrosis factor α (TNF-α) and IL-12 induced by toll-like receptor 2 (TLR2) (28, 29). Li et al. also found that in the patients with chronic HBV infection, monocytes expressed significantly higher levels of anti-inflammatory cytokines (TGF-*β* and IL-10) than those of healthy control. Furthermore, experiments *in vitro* showed that monocytes induced by hepatitis B surface antigen could educate NK cells to secrete IL-10 by mediating PD-L1/PD-1 and HLA-E/CD94 and inhibit autologous T cell activation (30). Our findings are consistent with the previous reports. Moreover, we identified a novel lncRNA-HEIM and verified its connection with TGF-*β* secretion in the CHB patients with long-term antiviral treatment, offering new evidence to the active role of lncRNA in the immune response upon HBV chronic infection.

Liver fibrosis is the common result of the liver’s healing response to chronic damage, characterized by an abnormal and excessive accumulation of the extracellular matrix (ECM) constituents (31). The main mediators of fibrosis are usually hepatic stellate cells (HSCs), which could be activated and transdifferentiated to myofibroblasts (MFBs) upon the liver’s inflammatory response and cytokines and secrete ECM proteins (32, 33). Among the cytokines, the TGF-*β* family, or TGF-*β*1 to be more specific, played a critical role in the development of hepatic fibrosis (34). Our data showed that increased TGF-*β* secretion from monocyte Thp-1 upon HBV infection could promote the fibrosis in HSCs at both mRNA and protein levels. More importantly, interfering the endogenous level of lncRNA-HEIM could significantly decrease the secretion of TGF-*β* and attenuate the fibrosis of HSCs. These findings provide new insights into the understanding and potential therapeutic strategies on chronic HBV infection; however, more work is needed to be done in the future to further demonstrate the detailed molecular mechanism between lncRNA-HEIM and TGF-*β* signaling pathway.

Evidence of the TGF-*β* autocrine loop in monocytes has been documented in the development and homeostasis of alveolar macrophages (35), in the proliferation, cell aggregation, and differentiation of human myelomonocytic U937 leukemia cells (36) (37), in myelofibrotic monocytes of patients with myelofibrosis (38), and so on. The downstream effectors of canonical TGF-*β* are SMADs including receptor-regulated SMADs (R-SMADs, *i.e.*, SMAD2 and SMAD3), common mediator SMAD4, inhibitory SMADs (I-SMAD, *i.e.*, SMAD6 and SMAD7) (39). As a critical effector of intracellular signaling, SMAD4 interacts with two R-SMAD molecules to form a heteromeric complex. This complex, presumably a trimer, is then translocated into the nucleus, where it activates transcription of defined genes. In our study, we found that HBV could significantly up-regulate the expression of SMAD4 at both mRNA and protein levels, affecting neither the total nor the phosphorylated R-SMADs. It indicated the possibility that altering the expression of SMAD4 only is sufficient for the activation of TGF-*β* signaling pathway. Meanwhile, we’ve noticed that there was a report indicating that HBV-encoded pX oncoprotein directly interacts with SMAD4, stabilizing the complex of SMAD4 with components of the basic transcriptional machinery and contributing to HBV-associated liver fibrosis (40). Li et al. also reported that SMAD4 could form a positive feedback signaling loop of TGF-*β*1-CD147 by direct interaction with CD147 promoter and modulating the active phenotype of HSCs, promoting liver fibrosis (25). These reports provided useful information for the following research of molecular mechanism of lncRNA-HEIM. Also, we’ve analyzed the lncRNA-HEIM interacting proteins by RNA pull-down assay followed by mass spectrometry and found several interesting candidates (data not shown). We are still verifying the authenticity of these lncRNA-interacting mRNAs. Currently, the detailed underlying molecular mechanism, including the involving role lncRNA-HEIM played during the process, is still unclear, and future research is needed to fully understand how it works upon HBV infection.

As one of the emerging research hotspots, lncRNAs are increasingly emphasized, especially on cancer research. As on fibrosis, accumulating evidence has revealed that lncRNAs could play both promotive and inhibitory roles in the process of multifaceted processes of fibrosis in various organs including the liver, heart, lung, and kidney (41). Wu et al. reported that up-regulation of lncRNA metastasis-associated lung adenocarcinoma transcript 1 (MALAT1) in liver fibrosis could activate hepatic stellate cells (HSCs) by suppressing silent information regulator 1 (SIRT1) (42). Yu et al. found that lncRNA Alu-mediated p21 transcriptional regulator (APTR) activated HSCs by negatively regulating the expression of p21, leading to the advancing of liver fibrosis in mouse model (43). Moreover, Yu et al. reported that lncRNA Homeobox transcript antisense RNA (HOTAIR) could enhance the methylation of phosphatase and tensin homolog deleted on chromosome 10 (PTEN) and contributed to the progression of liver fibrosis (44). However, most of these findings focused on the roles that lncRNAs played in either hepatocytes or HSCs. Here we laid eyes on monocytes, which are a smaller family of PBMCs, but vital actors in the immune-response against HBV infection. For the first time, the biological functions and potential molecular mechanism of lncRNA-HEIM during HBV chronic infection were documented. These findings increased the understanding of the roles lncRNAs played in the activation of HSCs and liver fibrosis in chronic HBV infection, providing a novel therapeutic target in the future treatment of chronic HBV infection.

## Data Availability Statement

The microarray data used in this research has been uploaded into GEO, with an accession number GSE166759.

## Ethics Statement

The studies involving human participants were reviewed and approved by the Ethics Committee of the First Affiliated Hospital, College of Medicine, Zhejiang University. The patients/participants provided their written informed consent to participate in this study.

## Author Contributions

JY, CL, and HD conceived and designed the study. CL, JJ, XZ, and TL performed the experiments. JY and FL analyzed and interpreted the data. JY and HD drafted the manuscript. JY, TL, and HD revised the manuscript. All authors contributed to the article and approved the submitted version.

## Funding

This work was supported by the Major National S&T Projects for Infectious Diseases (2018ZX10301401), Key Research & Development Plan of Zhejiang Province (2019C04005), and the National Key Research and Development Program of China (2018YFC2000500).

## Conflict of Interest

The authors declare that the research was conducted in the absence of any commercial or financial relationships that could be construed as a potential conflict of interest.
