# Supplementary figures and images for: lncRNA-HEIM Facilitated Liver Fibrosis by Up-Regulating TGF-β Expression in Long-Term Outcome of Chronic Hepatitis B
Source: Front Immunol. 2021 Jun 8;12:666370. doi: 10.3389/fimmu.2021.666370 (PMC8217658; doi:10.3389/fimmu.2021.666370)

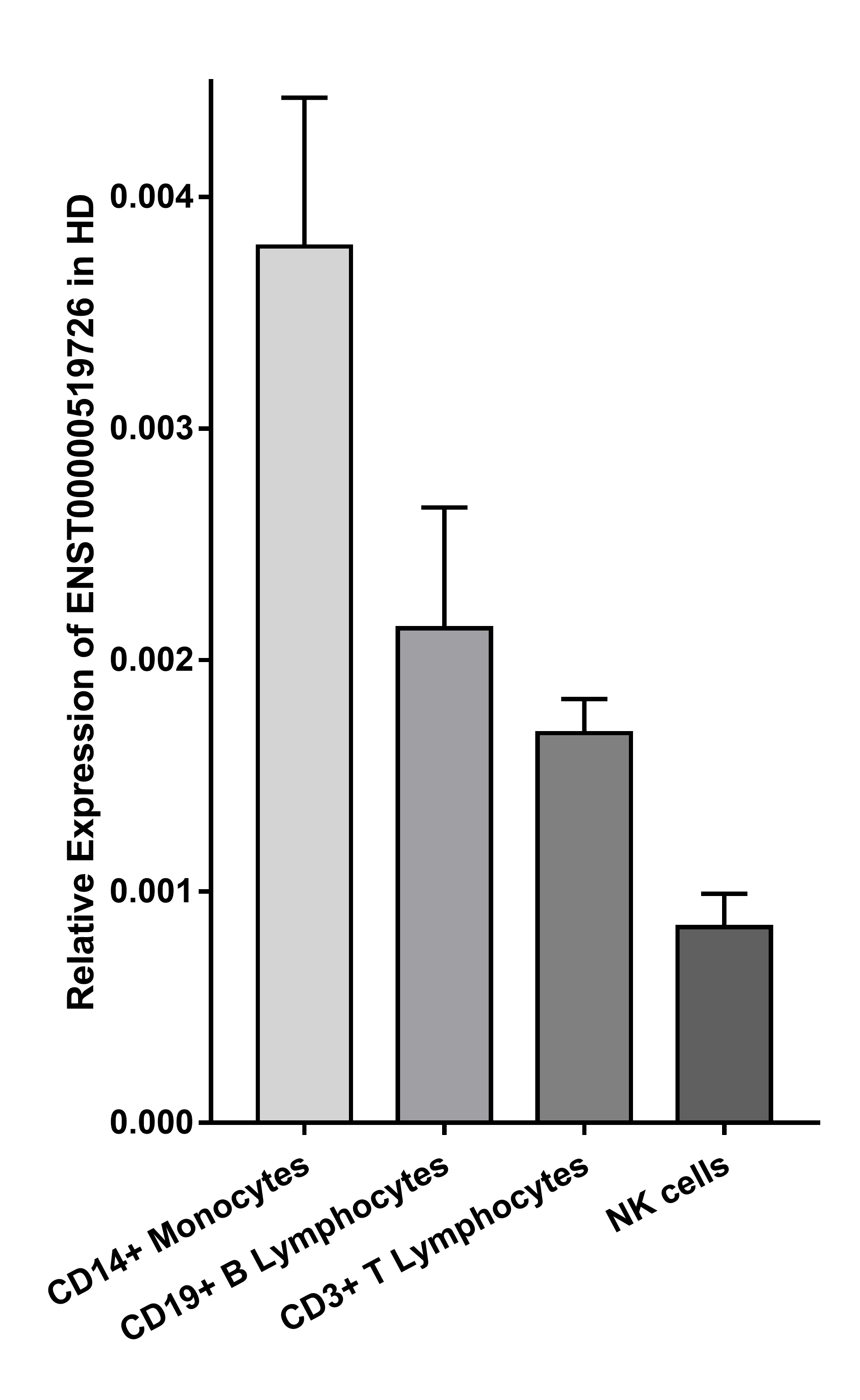

Supplement: Supplementary file 1 [file Image_1.tif]
